# Association between peripheral arterial occlusive disease and cardiothoracic ratio in patients on chronic hemodialysis

**DOI:** 10.1038/srep38458

**Published:** 2016-12-05

**Authors:** Kang-Yi Liou, Hung-Hsiang Liou, Yu-Wei Fang, Jyh-Gang Leu, Ming-Hsien Tsai

**Affiliations:** 1 Division of Nephrology, Department of Internal Medicine, Shin-Kong Wu Ho-Su Memorial Hospital, Taipei, Taiwan (R.O.C); 2 Division of Nephrology, Department of Internal Medicine, Hsin-Jen Hospital, New Taipei City, Taiwan (R.O.C); 3 Fu-Jen Catholic University School of Medicine, Taipei, Taiwan (R.O.C); 4 Division of Biostatistics, Institutes of Epidemiology and Preventive Medicine, College of Public Health, National Taiwan University, Taipei, Taiwan (R.O.C)

## Abstract

The cardiothoracic ratio (CTR) and peripheral arterial occlusive disease (PAOD) are related to mortality in hemodialysis patients. However, data on the association between PAOD and CTR are limited. In this study, we aim to elucidate this relationship in patients on chronic hemodialysis. Using a retrospective cross-sectional study of 622 Taiwanese patients, we investigated the association of PAOD and CTR. PAOD was significantly associated with CTR in the crude analysis. The odds ratio (OR) for CTR >0.5 was 1.77 [95% confidence interval (CI), 1.32–2.37], and the odds ratio for CTR >0.6 was 2.18 [95% CI, 1.44–3.30]. After adjusting for confounding variables, this difference continued to exhibit significant predictive power for CTR >0.6 (OR, 1.88; 95% CI, 1.14–3.11), but the predictive power for CTR >0.5 was attenuated (OR, 1.41; 95% CI, 0.98–2.03). In the subgroup analysis, PAOD was an independent factor for CTR >0.6, particularly in elderly and female patients or patients with hemoglobin >10 mg/dl and with no history of cardiovascular disease. In this research, we showed that the detection of PAOD was independently associated with CTR >0.6 in patients on chronic hemodialysis.

The cardiothoracic ratio (CTR) represents the left ventricular size and is estimated from chest X-rays as a proportion of the thoracic diameter^1^; furthermore, CTR is negatively associated with cardiac systolic dysfunction^2^. In general, the higher the CTR value, the larger the size of the heart. A CTR >50% is thought to represent cardiomegaly and is a prognostic factor in elderly populations^3^, patients with congestive heart failure^4^, and patients on dialysis^5^,^6^. Moreover, an enlarged heart increases the risk of fatal arrhythmia^7^. Therefore, CTR provides an easier representation of the status of cardiac remodeling and an easy method to assess the heart condition of chronic kidney disease (CKD) patients.

The ankle–brachial index (ABI), which is a ratio of ankle to brachial systolic blood pressure (BP), is a simple, non-invasive and reliable tool to diagnose peripheral arterial occlusive disease (PAOD)^8^, an atherosclerotic disorder that refers to varying degrees of occlusion of the lower limb arteries and is frequently observed in CKD^9^,^10^ and chronic hemodialysis (CHD) patients^11^,^12^. Furthermore, the ABI is a predictor of all-cause or cardiovascular mortality in CHD patients^12^,^13^,^14^,^15^,^16^. A lower (<1.0) or higher (>1.4) ABI value in CKD patients induces a higher event rate of acute myocardial infarction or cardiovascular disease (CVD)^17^.

Both cardiomegaly and PAOD contribute to CVD development in CHD patients. However, little evidence is available to evaluate the association of PAOD with CTR in patients with CHD. Akasawa *et al*. reported that PAOD had no significant association with CTR >0.5^18^. However, their PAOD diagnosis was assessed based on medical history alone and may have underestimated PAOD prevalence. We postulated that PAOD would complicate cardiac enlargement, which indicates cardiac remodeling in patients with CHD. This study investigates the association between PAOD and CTR in patients with CHD.

## Methods and Materials

### Study design and patients

**Figure 1:**
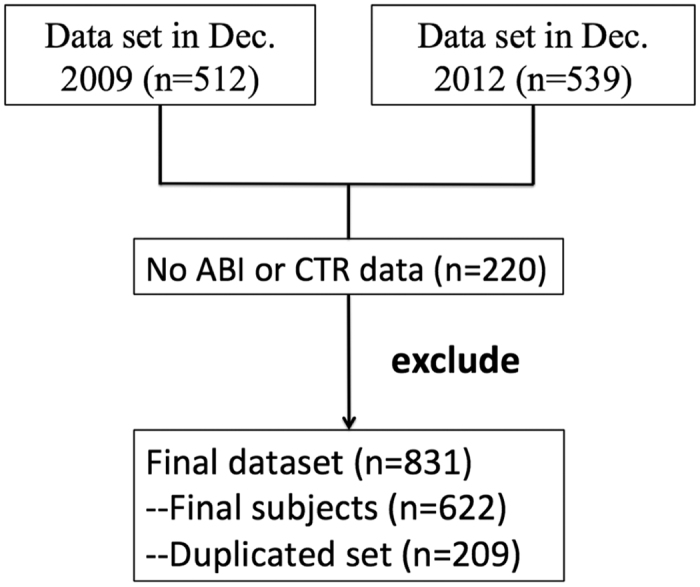
Schema of patient enrollment in the study.

This retrospective cross-sectional study was conducted at a single medical center. To be eligible for this study, patients must have undergone regular hemodialysis (HD) for at least 3 months before inclusion. Patients were required to have been clinically stable for 3 months prior to the study; specifically, patients with acute cardiovascular event cerebrovascular disease, infection, or other active diseases were excluded. Finally, a total of 622 patients on regular HD in the dialysis unit of Shin Kong Wu Ho-Su Memorial Hospital from December 2009 to December 2012 were included in the study (Fig. 1). This study was performed in accordance with the principles of the Declaration of Helsinki and was approved by the ethics committee of Shin-Kong Wu Ho-Su Memorial Hospital. Informed consent was waived because our study was based on medical chart review. Patient information was anonymized and de-identified prior to analysis.

### Medical and laboratory data

Demographic and medical data were obtained from the patients’ medical records upon entry into the study and included age; gender; smoking history (never vs. ever); blood pressure (BP); history of diabetes mellitus (DM), hypertension, coronary artery disease, or cerebrovascular disease; body mass index (BMI; weight/height^2^); duration on HD, CTR, and ABI; co-morbid conditions; intake of renin-angiotensin system (RAS) blockers, statins, beta-blockers, and anti-platelet agents; serum levels of blood urea nitrogen (BUN), creatinine (Cr), albumin, uric acid, total cholesterol (TC), triglyceride (TG), iron profile, hemoglobin (Hb), intact parathyroid hormone (iPTH), ionized calcium (iCa), and phosphate (P); and urea kinetics (Kt/V), determined according to the procedure described by Shinzato *et al*.^19^ CVD was diagnosed according to documented histories of coronary artery or cerebrovascular disease. Blood samples were collected after at least 8 hours of fasting and before the dialysis session.

### Cardiothoracic ratio measurement

At the end of the year at our medical center, posterior-anterior chest radiographs were routinely obtained after HD sessions in patients with CHD to measure the CTR. Computer assistance was employed to ensure accurate measurement. A reference vertical line was drawn on the radiograph through the midpoint of the spine from the sternum to the diaphragm. The maximum transverse diameter of the heart was obtained by adding the widest distance at the midline from the right to the left heart borders. Thoracic width was measured as the distance between the inner aspects of the widest points of the rib cage. The CTR was determined by dividing the maximal horizontal width of the heart by the horizontal inner width of the rib cage. Therefore, a CTR >50% was defined as cardiomegaly; a higher CTR indicated increased severity of cardiomegaly.

### Ankle brachial index

At our medical center, a PAOD survey for ABI measurement was performed in patients with CHD from 2009 and 2012. ABI was measured using a sphygmomanometer and a sphygmograph device during the HD session. BP was measured in all patients after a minimum rest of 5 minutes: cuffs with pressure sensors were wrapped around the arm without vascular access and around both ankles. The systolic BP of the brachial pulses was recorded for the upper limb. To measure the systolic BP of the dorsalis pedis and posterior tibial arteries in the lower limbs, the BP cuff was applied proximal to the ankle, inflated rapidly and deflated gradually. The mean of these two readings was used as the ankle systolic BP. The ABI was calculated by dividing the ankle systolic BP by the brachial artery systolic BP. The systolic BP of the arm without dialysis access and the bilateral ankle pressure were used separately for the calculation. Patients with an ABI of <0.9 in either leg were considered to have varying degrees of PAOD in their lower extremities.

### Statistical analyses

Data were expressed as the mean ± standard deviation (SD) or median (25^th^, 75^th^ percentile), as appropriate for continuous or categorical variables. Independent *t*-tests were used to compare the means of continuous variables, and the chi-square test was used for categorical variables. Moreover, a generalized mixed linear model was used to determine the risks of CTR >0.5 and CTR >0.6 with a link function of logit. We modeled the variance-covariance of residuals for repeated measurements as first order autoregression [AR (1)]. Using a modified stepwise procedure with five modeling steps, a separate regression model was used for dichotomous CTR as a function of PAOD. We also performed subgroup analysis including factors such as DM, age (≤65 years and >65 years), gender, previous CVD and hemoglobin level (≤10 g/dL and >10 g/dL). A *P value* ≤ 0.05 was considered statistically significant. All statistical analyses were performed using SAS for Windows version 9.3 (SAS Institute Inc., Cary, NC, USA).

## Results

**Figure 2:**
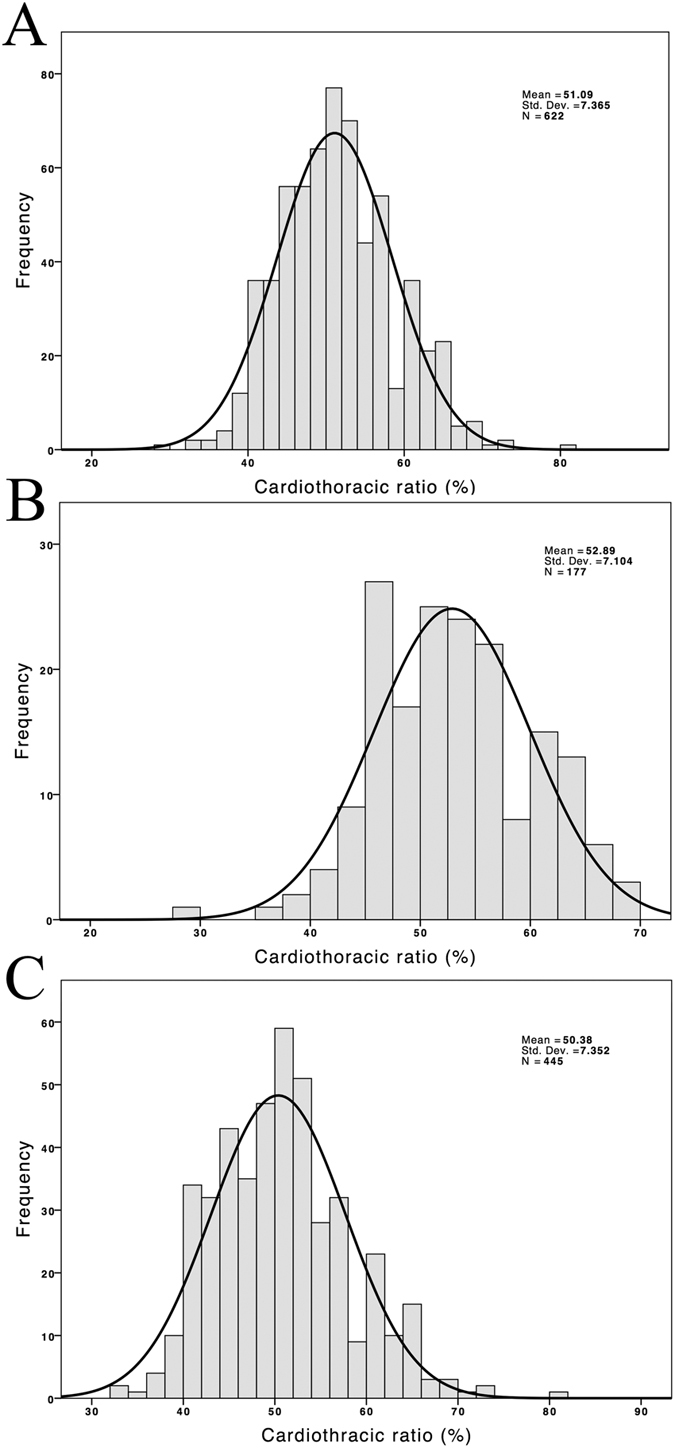
Distribution of cardiothoracic ratio values in all participants (**A**) patients with PAOD (**B**) and patients without PAOD (**C**).

**Figure 3:**
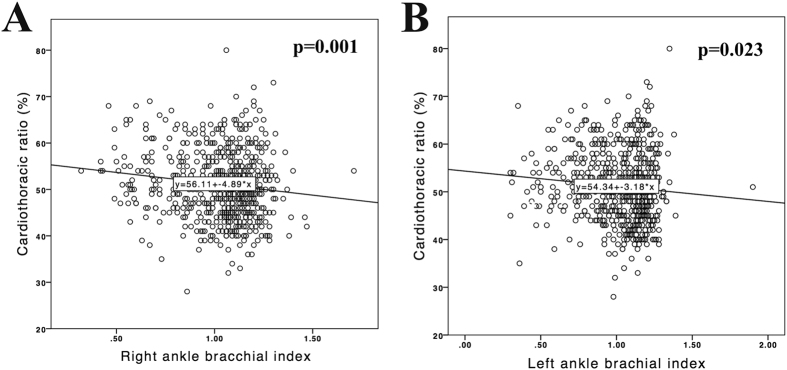
Regression plot of cardiothoracic ratio (%) (**A**) with right ankle brachial index and (**B**) with left ankle brachial index. Lines indicate best-fit regression lines derived from the least mean square method.

The 622 CHD patients had a mean age of 62.7 ± 13.5 years and had been on HD for a mean duration of 6.9 ± 5.3 years. Among them, 40.7% were diabetic, and 49.7% were men. CTR was >0.5 in 308 (49.8%) patients and >0.6 in 74 (11.9%) patients. The mean CTR was 51 ± 7.4% (25^th^–75^th^ percentile, 46–56%), as shown in Fig. 2. Moreover, a significant linear association was observed between CTR and ABI on the right side and the left side (Fig. 3). In total, 177 (28.4%) patients were diagnosed with PAOD based on an ABI of <0.9 in either leg. The other clinical characteristics of the participants are presented in Table 1.

### PAOD and non-PAOD

Table 1 also lists the demographic and clinical data of the participants stratified by the presence of PAOD. Patients with PAOD had significantly older age and increased Kt/V and CTRs, in either dichotomous or continuous form; reduced albumin, P and BP; and a reduced incidence of previous CVD and DM (all *P value*s, <0.05). No significant difference was noted regarding gender, HD duration, smoking status, medication use, BMI, iron profile, lipid profile, Hb, iPTH and iCa among the subgroups dichotomized by PAOD (all *P* values, <0.05).

### Determinants of CTR >0.5 and CTR >0.6

Factors associated with CTR >0.5 and >0.6 were assessed by a generalized mixed model with link function of logit. Table 2 shows that in the crude analysis, age, gender, DM, previous CVD, smoking status, diastolic BP, BMI, albumin, Hb, transferrin saturation, P and PAOD were significantly associated with CTR >0.5 (all *P value*s < 0.05). After adjusting for multiple variables, only age [odds ratio (OR), 1.04; 95% confidence interval (CI), 1.02–1.06], gender (OR, 0.59; 95% CI, 0.39–0.89), previous CVD (OR, 1.75; 95% CI, 1.22–2.50), BMI (OR, 1.06; 95% CI, 1.01–1.10), TG (OR, 0.99; 95% CI, 0.99–1.00), Kt/V (OR, 0.33; 95% CI, 0.14–0.76), Hb (OR, 0.81; 95% CI, 0.70–0.94) and transferrin saturation (OR, 0.97; 95% CI, 0.95–0.98) exhibited significant associations with CTR >0.5.

In the crude analysis, age, gender, previous CVD and DM, smoking status, albumin, Hb, transferrin saturation, and PAOD were significantly associated with CTR >0.6 (Table 3). After adjusting for multiple variables, only age (OR, 1.05; 95% CI, 1.02–1.07), previous CVD (OR, 1.68; 95% CI, 1.02–2.77), smoking (OR, 0.25; 95% CI, 0.11–0.62), transferrin saturation (OR, 0.96; 95% CI, 0.94–0.98) and PAOD (OR, 1.88; 95% CI, 1.14–3.11) remained significantly associated with CTR >0.6.

### Association between PAOD and CTR

On further crude analysis, PAOD was a significant risk factor for CTR 0.5–0.6 (OR, 1.52; 95% CI, 1.10–2.08) and CTR >0.6 (OR, 2.18; 95% CI, 1.44–3.30). However, after adjusting for the multiple variables age, gender, HD duration, DM, previous CVD, smoking status, BP, albumin, TG, TC, BMI, Hb, iPTH, ferritin, TSAT, KT/V, iCa, P and intake of anti-platelet, RAS blockers, beta blockers and statins, the ability of PAOD to predict a CTR of 0.5 to 0.6 was attenuated (OR, 1.24; 95% CI, 0.85–1.81) but remained significant for CTR >0.6 (OR, 1.88; 95% CI, 1.14–3.11) (Table 4).

### Subgroup analysis of CTR >0.6

**Figure 4:**
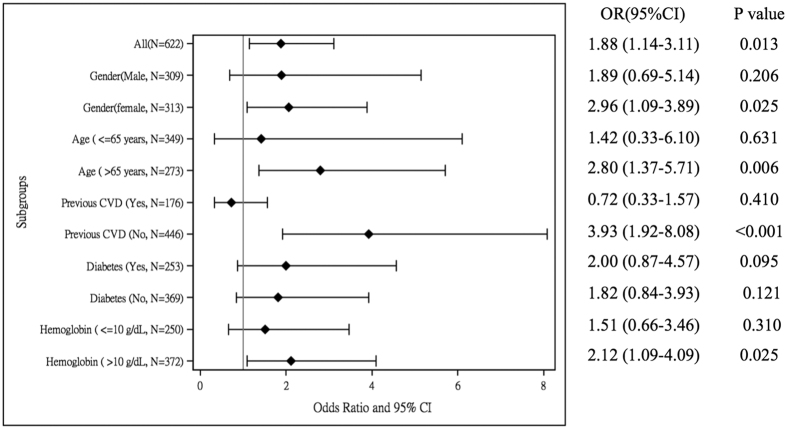
Subgroup analysis of the effect of PAOD on cardiothoracic ratio >0.6. The full model comprised adjusted variables, including age, gender, hemodialysis duration, diabetes mellitus, previous cardiovascular disease, smoking status, blood pressure, albumin, triglyceride, total cholesterol, body mass index, hemoglobin, intact parathyroid hormone, ferritin, transferrin saturation, urea kinetics, ionized calcium, phosphate and medications.

We investigated the association between PAOD and CTR >0.6 in analyses stratified by covariates, including history of DM and previous CVD, Hb (>10 g/dl and ≤10 g/dl), age (<60 years and ≥60 years) and gender. Figure 4 shows that after multivariate adjustment for demographic characteristics and dialysis-related chemistry data and medications, PAOD had a significant predictive power for CTR >0.6 in older patients (OR, 2.80; 95% CI, 1.37–5.71), female patients (OR, 2.96; 95% CI, 1.09–3.89), patients without CVD history (OR, 3.93; 95% CI, 1.92–8.08) and patients with Hb > 10 mg/dl (OR, 2.12; 95% CI, 1.09–4.09).

## Discussion

In this cross-sectional study of 622 patients with CHD, PAOD was independently correlated with CTR, especially with CTR >0.6. The correlation between PAOD and CTR >0.6 was independent of traditional anemia risk factors and dialysis quality. Moreover, in the subgroup analysis, this association was significant in the elderly, women and patients without CVD history and with Hb > 10 mg/dl. The results of this study could provide physicians with evidence to manage PAOD and prevent further cardiac enlargement during the dialysis of end-stage renal disease (ESRD) patients.

After adjusting for multiple variables in our CHD patients, we hypothesized that PAOD was significantly associated with severe cardiomegaly (CTR >0.6) but not with CTR >0.5. There are several co-morbidities in dialysis patients, such as DM, hypertension, anemia, malnutrition and chronic inflammation; fluid status may also contribute to cardiomegaly^20^,^21^,^22^. Moreover, reduced ABI is associated with PAOD^23^,^24^ and secondary to DM, smoking, hypertension, advanced age and hyperlipidemia^25^. These results were comparable with our data. Therefore, mild cardiomegaly and PAOD may develop concurrently due to similar risk factors. However, peripheral arterial occlusion may further induce a pumping load to the heart, which may eventually contribute to cardiomegaly progression. Therefore, it is reasonable to think that PAOD might play a major role in the exacerbation but not in the initiation of cardiomegaly. This finding implied that preventing PAOD development in dialysis or even in CKD patients would halt further cardiac remodeling. However, this idea requires further study to validate the effect.

CVD is a common co-morbidity that contributes significantly to the death rate in patients with ESRD^26^,^27^. In clinical practice, CTR is an easy and quick method of assessing a patient’s heart condition. Various previous studies have demonstrated that the CTR had a considerable impact on predicting mortality in patients on HD, and it was suggested to be a first-line approach to evaluate the presence of cardiac disease in a patient with CHD^5^,^24^,^28^. In this study, the factors that determined CTR >0.6 in CHD patients were identified after adjusting for multiple variables, including old age, previous CVD history, non-smoking status and lower transferrin saturation. Increased vascular stiffness at older ages is independently associated with severe cardiomegaly^29^. Smoking is negatively associated with severe cardiomegaly, potentially due to the enlarged chest cavity of smokers. Therefore, we did not consider smoking to be a protective factor against cardiomegaly development. In this study, Hb level was not associated with severe cardiomegaly, but transferrin saturation, which reflects iron storage, was an exacerbating factor in cardiomegaly progression. Interestingly, the association between PAOD and severe cardiomegaly was significant, especially in patients without anemia or CVD history. A reasonable explanation would be that anemia and CVD might be strong risks for cardiomegaly development. Therefore, the association of PAOD and severe cardiomegaly was attenuated in such groups. Regarding the gender difference, we hypothesized that estrogen or androgen might play an important role, but this idea requires further study for confirmation.

Our study had certain limitations. First, the study utilized a cross-sectional design. Therefore, causation between PAOD and cardiomegaly cannot be inferred. Based on pathophysiology, we hypothesize that vascular atherosclerosis developed initially and then led to further cardiomegaly. However, further cohort studies are needed to verify this causation. Second, we did not assess intra- or interobserver variability for the measurement of ABI and CTR. Nevertheless, ABI and CTR measurements are commonly and easily performed and are reliable. Finally, this work was a single-center study, which might not be applicable to all CHD populations. Nevertheless, the results of data analysis on the association between risk factors and CTR were compatible with the findings of previous studies^6^,^18^.

## Conclusion

PAOD was strongly related to severe cardiomegaly in CHD patients. This finding suggested that treating PAOD beyond the traditional approach might be beneficial in delaying the progression of cardiomegaly.

## Additional Information

**How to cite this article**: Liou, K.-Y. *et al*. Association between peripheral arterial occlusive disease and cardiothoracic ratio in patients on chronic hemodialysis. *Sci. Rep.* **6**, 38458; doi: 10.1038/srep38458 (2016).

**Publisher's note:** Springer Nature remains neutral with regard to jurisdictional claims in published maps and institutional affiliations.

## Figures and Tables

**Table 1:** Baseline characteristics of study population.

Characteristic	All (n = 622)	Non-PAOD (n = 445)	PAOD (n = 177)	*P value*
Age (yrs)	62.7 ± 13.5	60.4 ± 13.3	68.3 ± 12.2	< 0.001
Male sex (%)	309 (49.7)	231 (51.9)	78 (44.0)	0.091
Duration of dialysis (yrs)	6.9 ± 5.3	7.0 ± 5.4	6.6 ± 4.9	0.341
Diabetes mellitus (%)	253 (40.7)	154 (34.6)	99 (55.9)	<0.001
Previous CVD (%)	176 (28.3)	114 (25.6)	62 (35.0)	0.023
Smoking (%)	118 (19)	92 (20.6)	26 (14.6)	0.09
Systolic BP (mmHg)	146.2 ± 35.3	154.0 ± 31.1	127.5 ± 38.1	<0.001
Diastolic BP (mmHg)	77.5 ± 17.2	81.5 ± 15.6	67.8 ± 17.1	<0.001
Body mass index (kg/m^2^)	23.0 ± 4.06	23.0 ± 3.9	23.1 ± 4.2	0.823
Albumin level (g/dL)	4.14 ± 0.37	4.2 ± 0.3	4.0 ± 0.3	<0.001
Triglyceride (mg/dL)	160.4 ± 121.1	157.0 ± 126.3	169.8 ± 108.9	0.236
Cholesterol level (mg/dL)	172.4 ± 42.4	172.1 ± 40.9	173.4 ± 46.2	0.742
Kt/V	1.63 ± 0.23	1.62 ± 0.24	1.66 ± 0.20	0.035
Cardiothoracic ratio	0.51 ± 0.07	0.50 ± 0.07	0.52 ± 0.07	<0.001
>0.5 (%)	308 (49.8)	204 (45.8)	104 (58.7)	0.003
>0.6 (%)	74 (11.9)	43 (9.6)	31 (17.5)	0.008
Hemoglobin (g/dL)	10.3 ± 1.31	10.4 ± 1.3	10.3 ± 1.2	0.420
iPTH (pg/mL)	188.8 ± 198	194 ± 209	176 ± 172	0.256
Ferritin (μg/dL)	598 ± 675	603 ± 784	586 ± 273	0.755
Transferrin saturation (%)	34.7 ± 13.6	35.0 ± 13.7	34.0 ± 12.8	0.426
Ionized calcium (mg/dL)	4.6 ± 0.45	4.6 ± 0.4	4.6 ± 0.4	0.878
Phosphate (mg/dL)	5.15 ± 1.38	5.2 ± 1.3	4.9 ± 1.3	0.018
Use of medications				
Anti-platelet (%)	201 (32.3)	153 (34.3)	48 (27.1)	0.088
RAS blocker (%)	162 (26)	117 (26.2)	45 (31.6)	0.919
Beta-blocker (%)	215 (34.6)	153 (34.3)	62 (35.0)	0.926
Statin (%)	172 (27.7)	125 (28.0)	47 (26.5)	0.766

PAOD, peripheral arterial occlusive disease; CVD, cardiovascular disease; BP, blood pressure; KT/V, urea kinetics; iPTH, intact parathyroid hormone; RAS, renin-angiotensin system.

**Table 2:** Determinants of CTR > 0.5.

Parameter	Crude analysis		Multivariate analysis	
	OR (95% CI)	*P* value	aOR (95% CI)	*P* value
Age (per yr)	1.04 (1.03–1.05)	<0.001	1.04 (1.02–1.06)	<0.001
Male vs. female	0.56 (0.42–0.76)	<0.001	0.59 (0.39–0.89)	0.012
Duration of dialysis (per yr)	1.01 (0.99–1.04)	0.239	1.03 (1.00–1.07)	0.028
Diabetes mellitus	1.45 (1.07–1.95)	0.014	1.30 (0.88–1.91)	0.178
Previous CVD	2.16 (1.60–2.90)	<0.001	1.75 (1.22–2.50)	0.002
Smoking (ever vs. never)	0.63 (0.44–0.90)	0.013	0.73 (0.46–1.14)	0.171
Systolic BP (per 1 mmHg)	0.99 (0.99–1.00)	0.764	1.00 (0.99–1.01)	0.296
Diastolic BP (per 1 mmHg)	0.99 (0.98–0.99)	0.036	1.00 (0.98–1.01)	0.802
Body mass index (per 1 kg/m^2^)	1.04 (1.00–1.07)	0.012	1.06 (1.01–1.10)	0.005
Albumin level (per 1 g/dL)	0.43 (0.29–0.63)	<0.001	0.94 (0.57–1.57)	0.834
Triglyceride (per 1 mg/dL)	0.99 (0.99–1.00)	0.311	0.99 (0.99–1.00)	0.045
Cholesterol level (per 1 mg/dL)	1.00 (0.99–1.00)	0.968	1.00 (0.99–1.01)	0.083
Kt/V (per 1 unit)	0.75 (0.41–1.37)	0.351	0.33 (0.14–0.76)	0.010
Hemoglobin (per 1 g/dL)	0.78 (0.69–0.87)	<0.001	0.81 (0.70–0.94)	0.005
iPTH (per 1 pg/mL)	1.00 (1.00–1.00)	0.323	1.00 (0.99–1.00)	0.190
Ferritin (per 1 μg/dL)	1.00 (1.00–1.00)	0.393	1.00 (0.99–1.00)	0.633
Transferrin saturation (per 1%)	0.96 (0.95–0.97)	<0.001	0.97 (0.95–0.98)	<0.001
Ionized calcium (per 1 mg/dL)	1.19 (0.88–1.61)	0.241	0.97 (0.67–1.40)	0.881
Phosphate (per 1 mg/dL)	0.89 (0.80–0.98)	0.023	0.92 (0.81–1.04)	0.212
PAOD	1.77 (1.32–2.37)	<0.001	1.41 (0.98–2.03)	0.060
Use of medications				
Antiplatelet	1.29 (0.97–1.73)	0.076	1.36 (0.96–1.94)	0.080
RAS blocker	1.16 (0.88–1.55)	0.278	1.03 (0.72–1.46)	0.854
Beta-blocker	1.10 (0.83–1.45)	0.489	1.24 (0.87–1.75)	0.219
Statin	1.06 (0.79–1.42)	0.051	1.26 (0.88–1.79)	0.196

CTR, cardiothoracic ratio; aOR, adjusted odds ratio; CVD, cardiovascular disease; BP, blood pressure; KT/V, urea kinetics; iPTH, intact parathyroid hormone; PAOD, peripheral arterial occlusion disease; RAS, renin-angiotensin system.

**Table 3:** Determinants of CTR > 0.6.

Parameter	Crude analysis		Multivariate analysis	
	OR (95% CI)	*P* value	aOR (95% CI)	*P* value
Age (per yr)	1.04 (1.02, 1.06)	<0.001	1.05 (1.02, 1.07)	<0.001
Male vs. female	0.56 (0.42, 0.76)	<0.001	1.07 (0.61, 1.88)	0.798
Duration of dialysis (per yr)	1.01 (0.99, 1.04)	0.239	1.03 (0.98, 1.08)	0.196
Diabetes mellitus	1.45 (1.07, 1.95)	0.014	1.34 (0.78, 2.31)	0.277
Previous CVD	2.12 (1.40, 3.20)	<0.001	1.68 (1.02, 2.77)	0.038
Smoking (ever vs. never)	0.28 (0.12, 0.62)	0.002	0.25 (0.10, 0.64)	0.003
Systolic BP (per 1 mmHg)	1.00 (0.99, 1.00)	0.282	1.00 (0.99, 1.02)	0.094
Diastolic BP (per 1 mmHg)	0.99 (0.98, 1.00)	0.510	1.00 (0.98, 1.02)	0.839
Body mass index (per 1 kg/m^2^)	0.97 (0.92, 1.02)	0.269	0.97 (0.91, 1.03)	0.314
Albumin level (per 1 g/dL)	0.44 (0.26–0.74)	0.002	0.75 (0.35, 1.58)	0.450
Triglyceride (per 1 mg/dL)	0.99 (0.99, 1.00)	0.130	0.99 (0.99, 1.00)	0.180
Cholesterol level (per 1 mg/dL)	0.99 (0.99, 1.00)	0.540	1.00 (0.99, 1.00)	0.630
Kt/V (per 1 unit)	0.72 (0.30, 1.71)	0.462	0.40 (0.13, 1.26)	0.118
Hemoglobin (per 1 g/dL)	0.84 (0.71, 0.99)	0.041	0.86 (0.69, 1.07)	0.199
iPTH (per 1 pg/mL)	1.00 (1.00, 1.001)	0.143	1.00 (1.00, 1.00)	0.059
Ferritin (per 1 μg/dL)	1.00 (0.99, 1.00)	0.390	1.00 (0.99, 1.00)	0.727
Transferrin saturation (per 1%)	0.96 (0.94, 0.98)	<0.001	0.96 (0.94, 0.98)	0.001
Ionized calcium (per 1 mg/dL)	1.10 (0.71, 1.72)	0.646	0.95 (0.55, 1.64)	0.861
Phosphate (per 1 mg/dL)	1.00 (0.86, 1.16)	0.961	1.13 (0.94, 1.36)	0.190
PAOD	2.18 (1.44, 3.30)	<0.001	1.88 (1.14, 3.11)	0.013
Use of medications				
Antiplatelet	1.01 (0.64, 1.58)	0.959	1.02 (0.60, 1.73)	0.932
RAS blocker	1.05 (0.68, 1.64)	0.840	0.89 (0.53, 1.49)	0.666
Beta-blocker	0.87 (0.56, 1.36)	0.564	1.05 (0.61, 1.78)	0.853
Statin	0.98 (0.61, 1.56)	0.943	1.16 (0.68, 1.97)	0.581

CTR, cardiothoracic ratio; aOR, adjusted odds ratio; CVD, cardiovascular disease; BP, blood pressure; KT/V, urea kinetics; iPTH, intact parathyroid hormone; PAOD, peripheral arterial occlusion disease; RAS, renin-angiotensin system.

**Table 4:** Multivariate regression analysis of risk factors for binary cardiothoracic ratio levels.

	Effect of PAOD on CTR > 0.5 and ≤ 0.6 (n = 545)		Effect of PAOD on CTR > 0.6 (n = 622)	
	Odds ratio (95% CI)	P value	Odds ratio (95% CI)	P value
Unadjusted	1.52 (1.10, 2.08)	0.009	2.18 (1.44, 3.30)	<0.001
Model 1	1.25 (0.89, 1.74)	0.185	1.74 (1.13, 2.67)	0.011
Model 2	1.19 (0.83, 1.69)	0.333	1.94 (1.23, 3.05)	0.004
Model 3	1.17 (0.82, 1.69)	0.372	1.93 (1.22, 3.03)	0.004
Model 4	1.22 (0.83, 1.78)	0.298	1.87 (1.14, 3.08)	0.013
Model 5	1.24 (0.85, 1.81)	0.325	1.88 (1.14, 3.11)	0.013

Multivariate model 1 is adjusted for age, gender and hemodialysis vintage. Multivariate model 2 comprises model 1 as well as diabetes mellitus, previous cardiovascular disease, smoking, systolic blood pressure and diastolic blood pressure. Multivariate model 3 comprises model 2 as well as albumin, triglyceride, total cholesterol and body mass index. Multivariate model 4 comprises model 3 as well as hemoglobin, intact parathyroid hormone, ferritin, transferrin saturation, KT/V ionized calcium and phosphate. Multivariate model 5 comprises model 4 as well as medications. Abbreviations: PAOD, peripheral arterial occlusive disease, CTR, cardiothoracic ratio; CI, confidence interval.
